# Imaging primary prostate cancer with 11C-Choline PET/CT: relation to tumour stage, Gleason score and biomarkers of biologic aggressiveness

**DOI:** 10.2478/v10019-012-0034-y

**Published:** 2012-06-19

**Authors:** Ji Chen, Yong Zhao, Xin Li, Peng Sun, Muwen Wang, Ridong Wang, Xunbo Jin

**Affiliations:** 1 Department of Minimally Invasive Urology center, Provincial Hospital Affiliated to Shandong University, Jinan, People’s Republic of China; 2 Department of PET/CT Center, Provincial Hospital Affiliated to Shandong University, Jinan, People’s Republic of China; 3 Center for Addiction and Mental Health, University of Toronto, Toronto, Canada

**Keywords:** prostate cancer, aggressiveness, PET/CT, choline

## Abstract

**Background:**

As a significant overlap of 11C-Choline standardized uptake value (SUV) between prostate cancer and benign prostate hyperplasia (BPH) tissue, controversy exists regarding the clinical value of 11C-Choline PET/CT scan in primary prostate cancer. In this study, the SUVmax of the prostate lesions and the pelvic muscles were measured and their ratios (SUVmax-P/M ratio) were calculated. Then we evaluated whether the tracer 11C-Choline uptake, quantified as SUVmax-P/M ratio, correlated with tumour stage, Gleason score, and expression levels of several biomarkers of aggressiveness.

**Methods:**

Twenty-six patients with primary prostate cancer underwent 11C-Choline PET/CT. Tumour specimens from these patients were graded histopathologically, and immunnohistochemistry for Ki-67, CD31, androgen receptor (AR), Her-2/neu, Bcl-2, and PTEN were performed.

**Results:**

Both SUVmax and SUVmax-P/M ratio showed no significant difference between patients with tumour stage II and III, but significantly elevated in patients with tumour stage IV. SUVmax-P/M ratio was also significantly higher in lesions with Gleason score of 4+3 or higher versus less than or equal to 3+4. SUVmax-P/M ratio was found significantly correlated with expression levels of Ki-67 and CD31. In addition, a higher SUVmax-P/M ratio was demonstrated in Her-2/neu positive subgroup than negative subgroup. At the same time, Gleason score and expression levels of these biomarkers showed no significant association with SUVmax.

**Conclusions:**

Using the parameter SUVmax-P/M ratio, 11C-Choline PET/CT may be a valuable non-invasive imaging technology in the diagnosis of primary prostate cancer.

## Introduction

Prostate cancer is one of the most common malignancies in males and the second leading cause of death in American men.^1^ The incidence of prostate cancer increases directly with age; however, this tumour shows variable biologic behaviour, from a clinically silent, indolent intra-prostatic tumour to an aggressive malignancy and causes death in a relatively small proportion of men.^2^ Therefore, identifying aggressiveness early in the disease process could be beneficial for therapeutic decision making.^3^–^5^ The development of prognostic markers that can accurately predict outcome is crucial to identify patients who could benefit from aggressive therapy.

In recent years, positron emission tomography combined with computed tomography (PET/CT), which allows image fusion of metabolic and anatomical information, has been reported as a non-invasive, whole-body imaging modality commonly used in the evaluation of many neoplasms including prostate cancer.^6^,^7^ PET/CT can provide more information than conventional tumour seeking procedures. This technique shows molecular function and activity that are not available with conventional imaging such as ultrasound and magnetic resonance imaging. In fact, clinical studies performed on several malignant tumours other than prostate cancer have shown that hypermetabolic tumours usually have high tracer uptake and a poor prognosis.^8^–^13^

The aim of the present study was to evaluate whether the use of 11C-Choline PET/CT would provide a non-invasive metabolic parameter that is associated with the biologic aggressiveness of prostate cancer. Gleason score and tumour stage were selected for analysis. Several molecular biomarkers of key prostate cancer pathogenic pathways including Ki-67 (proliferation), Bcl-2(apoptosis), CD31 (angiogenesis), Her-2/neu (oncogene), PTEN (anti-oncogene), and AR (androgen receptor) were also involved in this study. These data would further confirm the association between 11C-Choline uptake and prostate cancer allowing the integration of this tool into clinical practice.

## Patients and methods

This study was approved by the institutional review board of Provincial hospital affiliated to Shandong University, and written consent was obtained from all participants. Twenty-six patients with initial diagnosis of prostate cancer between April 2007 and March 2010 at our center were included in this study. Patients were excluded from this study if they had received antiandrogen therapy before PET/CT. All patients underwent ultrasound-guided transrectal biopsy of their tumour and histologically proved carcinomas. To avoid false positive accumulation of choline caused by existence of inflammatory cells, scanning was performed before histological intervention. And the results of PET/CT scan were taken as reference for biopsy. Patients were staged based on the 2002 American Joint Committee Cancer Staging Manual.^14^

### 11C-Choline PET/CT

PET/CT studies were performed the same as previously described in detail.^15^ 11C-Choline was synthesized according to the solid-phase method in a modified commercial synthesis module (TRACElab, FXc; GE Healthcare). Five minutes after injection of 7.4 MBq/kg of 11C-Choline, PET images were acquired in the supine position over 2 bed positions from the upper pelvis through the midthigh. For patients with a serum prostate specific antigen (PSA) >10 ng/ml, whole body PET/CT imaging was performed (6 bed positions). The parameters of the multidetector helical CT scan were 140 kV, 80 mA, 0.8 seconds per tube rotation, slice thickness of 5mm, pitch of 6, and table speed of 11.25 mm/s. CT and PET images were matched and fused into transaxial, sagittal, and coronal images. The 11C-Choline maximal standardized uptake value (SUVmax) was determined using a circular (1 cm diameter) region of interest. The SUVmax of the prostate lesions (target) and the pelvic muscles (nontarget) were measured and their ratios (P/M) were calculated. The PET/CT findings were compared with histopathologic and immunohistochemical results.

### Immunohistochemistry

All samples were taken at the sites selected with direct reference to the PET/CT scan results. As tumour stage of our cohort ranged from II to IV, for the patients who could not receive radical resection, biopsies at the site of abnormal uptake in PET/CT scan were selected for analysis. For immunohistochemical staining, paraffin sections (5-μm thickness) on glass slides coated with poly-L-lysine were deparaffinized in turpentine, hydrated, and then placed in phosphate-buffered saline (PH 7.6). Antigen retrieval was performed by boiling for 15 minutes in 0.01mol/L citrate buffer (PH 6.0). Sections were treated with 3% hydrogen peroxide for 10 minutes to quench endogenous peroxidase activity, rinsed with deionized water, and washed with phosphate-buffered saline. The primary antibodies used included mouse monoclonal antibodies against Ki-67 nuclear antigen (MIB-1), CD31 (JC/70A), AR (AR441), Her-2/neu (CB11), Bcl-2 (Bcl-2-100), and PTEN (28H6). All these antibodies were purchased from ZSGBBIO (Beijing, China) and diluted at 1:50 dilution. Sections were incubated with the first antibodies overnight at 4°C in a moist chamber and then were washed three times with phosphate-buffered saline. Detection of the antibodies was performed with a non-biotin horseradish peroxidase detection system, PV9000 Polymer Detection System (ZSGB-BIO, Beijing, China). After incubation with the polyperoxidase-anti-mouse/rabbit IgG for 30 minutes at room temperature, the tissue sections were washed with phosphate-buffered saline. Diaminobenzidinetetrahydrochloridechromogen was added for 3 minutes at room temperature, which causes brown precipitate at the antigen site. Sections were counterstained with hematoxylin, dehydrated, and then coverslipped with permount mounting medium.

Each slide was scored by two independent observers who were blinded to the clinicopathological data of the patients. Approximately 600 neoplastic cells were evaluated on each slide. The results of Ki-67 immunohistochemistry were expressed as the percentage of tumour cells with positive nuclear staining. Expression of PTEN (nuclear staining) was considered to be positive if there was staining of area of the epithelial component of the tumour. Staining of AR (nuclear staining), Her-2/neu (membranous staining), and Bcl-2 (cytoplasmic staining) were scored by evaluating the percentage of staining positively and the intensity of staining. Staining intensity was defined as 0, negative or weak; 1, moderate; or 2, strong. The level of immunohistochemical staining of AR was scored semiquantitatively as the product of the staining intensity score and the percentage of tumour cells with positive staining.^16^ As to the staining level of Her-2/neu and Bcl-2, either moderate or strong staining intensity in >10% of tumour cells was considered as positive expression.^17^

To investigate microvessel density (MVD), the number of vessels was counted on a ×400 magnification field (0.15mm^2^) in areas of the most intense neovascularization marked by CD31 expression in vascular endothelium from each sample. As in a previous report, any single cell or spot that stained by the CD31 was counted as a vessel. MVD was expressed as the absolute number of microvessels per ×400 field for each case.^17^

### Statistical analysis

Statistical analysis was performed using SPSS for Windows 17.0 software (SPSS, Inc., Chicago, IL, USA). The relationship between SUVmax-P/M ratio and Gleason score, tumour stage, expression levels of Her-2/neu, Bcl-2, PTEN were evaluated using the Mann-Whitney rank sum test. Correlation between MVD, expression levels of Ki-67, AR and SUVmax-P/M ratio was analysed using Spearman’s rank correlation test. Probability values of <0.05 indicated a statistically significant difference.

## Results

Patients’ characteristics are listed in Table 1. The mean age of the patients was 67.1 years (range, 56–81 years). There were 10 patients with stage II, 5 patients with stage III, and 11 patients with stage IV. Fourteen patients had a Gleason score ≥7 (4+3). As initial treatment, 12 patients were treated with radical prostatectomy, 13 patients antiandrogen therapy, and 1 patient radiotherapy. The mean value of SUVmax of the primary tumours in 26 patients was 9.42 ± 6.70 (ranged from 1.69 to 24.65), whereas the mean SUVmax-P/M ratios was 4.33 ± 1.41 (ranged from 1.18 to 7.44). Using 2.3 (SUVmax-P/M raito) as a criterion for differentiating malignant from benign prostatic lesions in our cohort, the sensitivity of 11C-Choline PET/CT imaging was 88.6% (23/26). The mean value of Ki-67 labeling index and AR index was 10.4±13.1 (ranged from 0 to 48) and 86.5 ± 61.8 (ranged from 0 to 180). The mean rate of MVD as assessed by CD31 was 26.4 ± 11.8 (ranged from 7 to 48). Positive expression of Her-2/neu, Bcl-2 and PTEN was recognized in 6, 13, and 11 patients, respectively.

**FIGURE 1:**
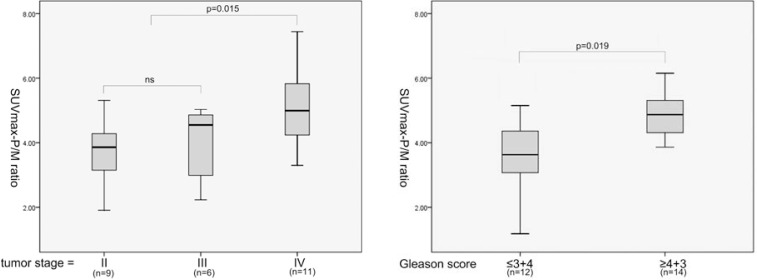
11C-Choline uptake (measured as SUVmax-P/M ratio) according to tumour stage and Gleason score. SUVmax-P/M ratio was significantly higher in patients with tumour stage or Gleason score > 7(3+4).

**FIGURE 2:**
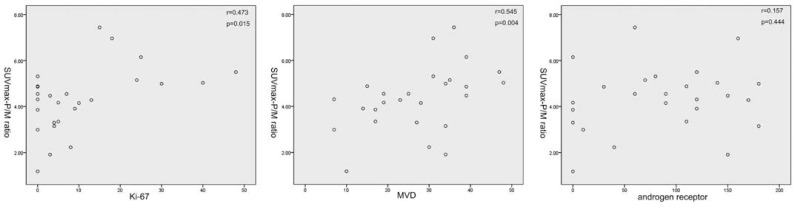
Correlation between 11C-Choline uptake (measured as SUVmax-P/M ratio) and expression of Ki-67, CD31 and AR.A statistically significant correlation was found between SUVmax-P/M ratio and expression levels of Ki-67 and CD31.

**FIGURE 3:**
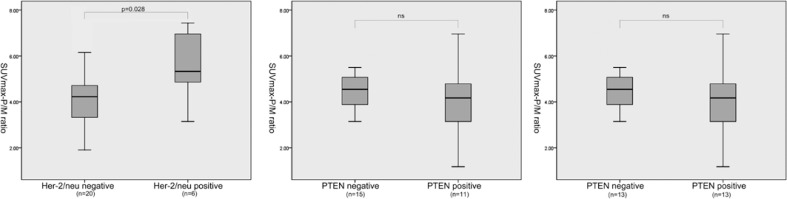
11C-Choline uptake (measured as SUVmax-P/M ratio) according to expression of Her-2/neu, Bcl-2, and PTEN. SUVmax-P/M ratio was significantly higher in patients with Her-2/neu positive staining.

Association of several clinicopathological factors and expression levels of these molecular markers was then analysed. The mean SUVmax in stages II, III, and IV were 5.88 ± 4.96, 10.69 ± 5.69, and 12.06 ± 7.50. The mean SUVmax-P/M ratio in stages II, III, and IV were 3.62 ± 1.26, 3.93 ± 1.25, and 5.16 ± 1.28. Both SUVmax-P/M ratio and SUVmax showed no significant difference between patients with tumour stage II and III (p-value 0.113 and 0.328, respectively), but significantly elevated in patients with tumour stage IV (p-value 0.036 and 0.015, respectively). Furthermore, SUVmax-P/M ratio was also significantly higher in patients with Gleason score of 4+3 or higher versus less than or equal to 3+4 (Figure 1). The correlation coefficients for SUVmax-P/M ratio with Ki-67 and MVD were 0.503 and 0.545, and the P values for both of these two biomarkers correlations were <0.05 (Figure 2). As shown in Figure 3, SUVmax-P/M raito was higher in Her-2/neu positive subgroup than negative subgroup. In contrast, neither Gleason score nor expression levels of the biomarkers involved in the study associated with SUVmax.

**FIGURE 4:**
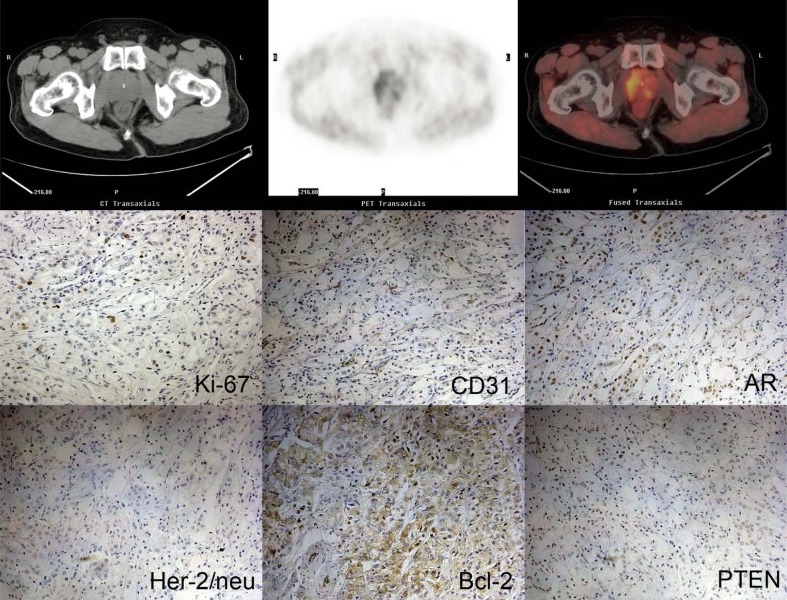
11C-Choline PET/CT images and immunohistochemistry images form patient No. 17.

**FIGURE 5:**
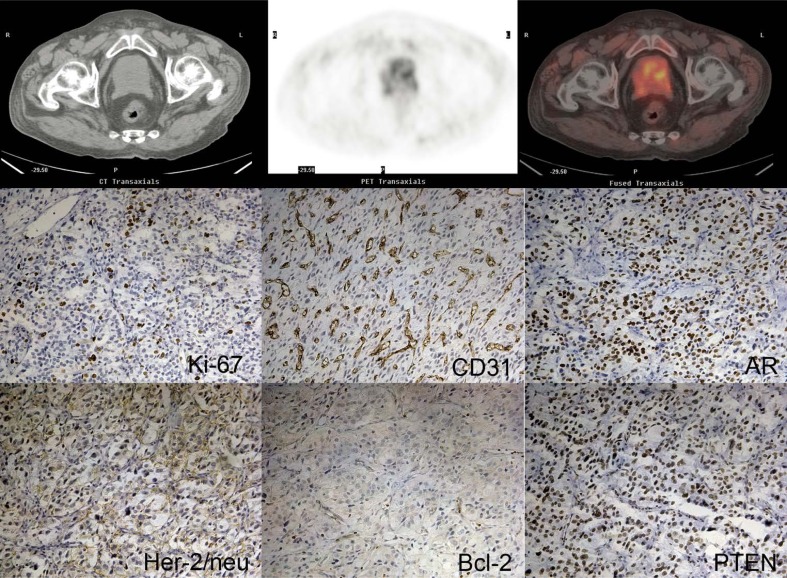
11C-Choline PET/CT images and immunohistochemistry images form patient No. 1.

Figure 4 and Figure 5 showed PET/CT imaging and immunohistochemistry results from two patients (patient 17 and patient 1). Patient 1, with tumour stage IV and a Gleason score of 7(4+3), demonstrated relatively higher expression levels of Ki-67, CD31, AR, PTEN and Her-2/neu. However, a higher SUVmax was found in patient 17 (7.58 versus 6.42). At the same time, uptake of 11C-Choline in patient 17 was also higher than in patient 1 (2.26 versus 1.17), which resulted in a relatively lower SUVmax-P/M ratio in patient 17 (3.35 versus 5.50).

## Discussion

PET is a whole-body non-invasive imaging technique that has great diagnostic value because it can identify damage by counting metabolic activity which is not available with other conventional imaging modalities. PET/CT, which combines a morphological imaging technique with a metabolic diagnostic technique, allows image fusion of metabolic and anatomical information and shows increasing usefulness in clinical oncology for disease staging, prognostic stratification, therapy planning, monitoring treatment and early detection of recurrence. The most used radiotracer in oncology, 18F-Fluorodeoxyglucose (18F-FDG), has shown to be an accurate tracer for tumour detection in many types of malignant tumours including breast cancer, colorectal cancer, esophageal cancer, melanoma, lung cancer, liver cancer, Wilms’ tumour, and sarcoma.^6^ However, use of this tracer is disappointing in detection of prostate cancer because of its physiologic urinary excretion and the low glucose metabolism of prostate cancer cells.^19^

Due to the limitation of 18F-FDG, alternative tracers for molecular imaging of prostate cancer have been introduced. Choline is a substrate for the synthesis of phosphatidylcholine, which is an essential component of all cell membranes. As carcinogenesis is characterized by enhanced cell proliferation that lead to increased membrane or fatty acid demands, the uptake of choline could reflect proliferative activity. Thus, choline labelled with 11C or 18F has been introduced for molecular imaging of prostate cancer. 11C-Choline PET/CT has been reported to give clear images of the pelvic region, prostate cancer, and pelvic lymph node metastases due to lower urinary excretion, while 18F-Choline PET/CT is more useful for possible distribution to centers lacking in on-site cyclotron.^20^

The diagnostic value of 11C-Choline PET/CT in imaging prostate cancer with increasing PSA after first-line treatment has been investigated by several authors, and this technique demonstrated to be more helpful than conventional imaging modalities in the detection of lymph node and distant metastases.^21^–^27^ However, its use for primary prostate cancer remains controversial. The results of different studies varied considerably. Some authors reported a significant overlap of 11C-Choline SUVmax between prostate cancer and benign prostate hyperplasia (BPH) tissue^22^,^27^, while some other studies showed that 11C-Choline PET was effective in differentiating malignant from benign prostate lesions.^28^–^31^

In our previous study on the usefulness of 11C-Choline PET/CT in detecting prostate cancer in patients with an elevated serum PSA level, we also found some BPH patients with rather higher prostate SUVmax. Moreover, it seemed that high SUVmax in these patients were usually accompanied with high muscle SUVmax. On the other hand, SUV methodology is affected by many factors such as patient size, time between tracer injection and PET/CT scan. So we calculated SUVmax-P/M ratios in all patients and the subsequent results demonstrated that there was a significant statistical difference in SUVmax-P/M raitos between malignant and benign lesions of prostate. Using 2.3 (SUVmax-P/M ratio) as a criterion, 11C-Choline PET/CT showed a sensitivity of 90.48%, a specificity of 85.71%, an accuracy of 87.76%, a positive predictive value of 82.61%, and a negative predictive value of 92.31%.^14^

As PET/CT is of limited clinical value if used only as a tumour seeking procedure, this study was conducted to evaluate the relationship between clincopathological parameters, molecular biomarkers of aggressiveness and 11C-Choline PET/CT imaging. In fact, several investigators also focused their attention on the relationship between 11C-Choline uptake in PET scans and biologic behaviour of primary prostate cancer in the last few years. However, like the usefulness of 11C-Choline PET in detecting primary prostate cancer, the association between 11C-Choline uptake and tumour biologic behaviour remains confusing. Sutinen *et al.* reported that no correlation could be demonstrated between the tumour uptake of 11C-Choline and the histological grade, Gleason score, volume of the prostate or PSA.^32^ These data were confirmed by Farsad *et al.*,^27^ and Giovacchini *et al..*^22^ Yamaguchi *et al.* observed significant linear correlations between SUVmax and serum PSA, while no significant correlations were found between SUVmax and Gleason score, or between SUVmax and tumour grade.^33^ Reske *et al*.,^31^ in agreement with another study by Breeuwsma *et al*.,^34^ reported that tumoral 11C-Choline uptake was related to tumour stage and no correlation was found between SUV and either the Gleason score or pre-operative serum PSA.^,^ In addition, Breeuwsma *et al*. compared the uptake of 11C-Choline with the amount of Ki-67 staining and demonstrated no relationship.^34^

However, it seems that these studies were restricted to the exclusive evaluation of apparent tumour SUV (mean or maximum). As a significant overlap of 11C-Choline uptake in PET imaging between malignant and benign lesions of prostate, the parameter SUV alone may not serve as a marker of tumour biologic behaviour. According to a more recent study by Piert *et al*. 35, in which the radioactivity concentrations of the tumour (T) and benign prostate background (B) were measured and their ratios (T/B) were calculated, high Gleason score (≥4+3) and Ki-67 index (≥5%) were significantly associated with an increased SUV T/B ratio in 11C-Choline PET/CT imaging. However, as the author mentioned in the study, it is difficult to define the true location of benign prostate tissue, which serve as radioactivity background.

In the light of our previous study, SUVmax-P/M ratio was added to express 11C-Choline uptake in the present study. And this parameter seems to be much more valuable than SUVmax in 11C-Choline PET/CT imaging of prostate cancer. Our results indicated that 11C-Choline SUVmax-P/M ratio was significantly associated with clinical tumour stage and Gleason score. The Gleason system is the most widely used grading of prostate carcinoma that ranges from 1 (well differentiated) to 5 (poorly differentiated). The Gleason score is the sum of the most prevalent and second most prevalent grade and prostate cancer could be classified as well and moderately differentiated (Gleason score 2–7) and poorly differentiated (8–10).^36^ A low Gleason score is associated with a more indolent malignancy with a good prognosis whereas a high Gleason score is indicative of an aggressive biologic behaviour and an increased risk of occult systemic disease.^37^ As there were 11 patients with a Gleason score of 7 in our cohort, we chose 7(3+4) as the cut-off Gleason score in the analysis and demonstrated a significantly elevated SUVmax-P/M ratio in poorly differentiated prostatic carcinoma.

The molecular biomarkers involved in this study included Ki-67, CD31, AR, Her-2/neu, Bcl-2, and PTEN. Among these markers, Ki67, CD31 and Her-2/neu showed association with SUVmax-P/M ratio, whereas expression levels of the remaining 3 proteins showed no association. Our results might suggest an association between aggressiveness of prostate cancer and upregulation of choline metabolism in tumour tissue. The nuclear antigen Ki-67 is a well-established molecular marker of proliferation in prostate cancer. As aggressive tumours have a higher proliferation rate compared with insignificant tumours, indicators of proliferation may be expected to be good predictors of clinical outcome. Several studies have already suggested that Ki-67 is a useful marker by significantly correlating with biologic aggressiveness and prognosis in prostate cancer.^15^,^38^–^40^ Our results, in agreement with the study by Piert *et al*.,^35^ demonstrate that 11C-Choline uptake measured by PET/CT could reflect proliferative activity of prostate cancer. CD31 is a pan-endothelial marker that can be used as a marker for the determination of vascular density. Angiogenesis is a fundamental process by which new blood vessels are formed, and it is essential for tumour growth by providing nutrients and eliminating metabolic waste products. It has been proposed as a promising prognostic marker in prostate cancer by several investigators.^41^–^42^ Our results demonstrated angiogenesis may promote 11C-Choline uptake by enhanced tracer delivery. As to Her-2/neu, since Slamon *et al*.,^43^ firstly reported that its overexpression was associated with high risk of relapse and death in breast cancer, the role of the Her-2/neu oncogene in prostate cancer progression and metastasis has been investigated. Her-2/neu expression is reported associated with unfavourable tumour phenotype, rapid tumour cell proliferation, PSA biochemical recurrence and poor prognosis.^44^–^45^ In addition, a higher expression level of Her-2/neu was reported in hormone-refractory prostate cancer.^46^–^47^ A study by Craft N *et al.* demonstrated that Her-2/neu overexpression provides an alternative mechanism for the activation of AR signalling pathways with low levels of testosterone, thereby contributing to androgen independent growth.^48^ It seems that expression rate of this biomarker in the present study was rather higher than the results reported by the researchers mentioned above. However, most of these studies were performed on radical prostatectomy specimens, while our cohort included patients with advanced prostate cancer who were not suitable for radical surgery. In fact, positive expression of Her-2/neu was recognized in 4 of 9 patients with tumour stage IV, while only 2 of 12 patients with relatively early stage. This may contribute to a higher SUVmax-P/M ratio in Her-2/neu positive subgroup, as this parameter was significantly correlated with tumour stage in our cohort.

This study has certain limitations. Firstly, the results from the small sample size will be questionable. A larger study with long-term follow-up in subgroups receiving different therapy would be necessary to determine directly whether SUVmax-P/M ratio is independent with respect to other well-known clinical and biologic prognostic variables in prostate cancer. Secondly, despite we selected prostate samples with reference to PET/CT results; we do not know for certain whether the specific tumour assessed by immunohistochemistry and pathology was accurately identified on imaging. Our study was not about assessing tumour localization by PET/CT. Nevertheless, our data showed that as the SUV-max P/M ratio increased, so did level of clincopathological and molecular markers in the representative tumour lesion assessed. Another limitation is that specimens in our study were obtained through different methods. And for the patients obtaining samples through biopsy, only those with an adequate specimen for immunohistochemical analysis could be included. These may bias to verify the results of our study. In addition, as some patients’ serum PSA tests were not performed at our center, we did not take this parameter into analysis. Further studies are needed to investigate the relationship between these parameters and 11C-Choline uptake.

## Conclusions

This pilot study indicates that the 11C-Choline uptake in primary prostate cancer normalized by pelvic muscle does correlate with Gleason score, tumour stage and expression of several tumour biomarkers. These findings confirm that PET/CT could be used as a non-invasive diagnostic modality that can provide information currently available only through pathological examination of tumour tissues. Because Gleason score and biomarkers involved in the study are prognostic markers of prostate cancer, the SUVmax-P/M ratio determined by 11C-Choline PET/CT may offer important biological information about prostate cancer. We might be able to pretherapeutically evaluate the biologic aggressiveness of primary prostate cancer by means of 11C-Choline PET/CT scan, and monitoring the SUVmax-P/M ratio would be informative for work-up and management of primary prostate cancer.

## Figures and Tables

**TABLE 1:** Clinicopathological characteristics of 26 patients with prostatic carcinoma

**No.**	**Age**	**GS**	Stage^*^	**SUVmax**	**SUVmax-P/M ratio**	**Initial treatment**
1	77	7	IV	6.42	5.5	Antiandrogen therapy
2	74	6	II	3.25	1.91	Radical resection
3	60	7	III	4.86	2.99	Radical resection
4	79	9	II	19.10	5.31	Antiandrogen therapy
5	68	7	IV	4.66	3.30	Antiandrogen therapy
6	69	7	II	5.26	4.15	Radical resection
7	70	5	II	3.31	1.18	Radical resection
8	59	7	III	5.05	4.55	Radical resection
9	63	8	IV	6.49	4.88	Antiandrogen therapy
10	56	9	IV	21.05	6.15	Antiandrogen therapy
11	63	7	II	4.01	4.28	Radical resection
12	63	8	IV	8.23	4.31	Antiandrogen therapy
13	81	7	III	11.58	5.03	Antiandrogen therapy
14	59	9	IV	9.38	7.44	Antiandrogen therapy
15	68	7	IV	24.65	5.15	Antiandrogen therapy
16	68	7	II	5.11	4.47	Radical resection
17	62	6	II	7.58	3.35	Radical resection
18	59	5	IV	4.49	6.96	Antiandrogen therapy
19	63	9	III	14.15	2.23	Radical resection
20	59	9	II	6.39	4.55	Radical resection
21	76	7	IV	8.26	4.99	Antiandrogen therapy
22	64	7	II	1.69	3.15	Radical resection
23	78	7	III	17.83	4.86	Radiotherapy
24	66	5	IV	19.96	3.91	Antiandrogen therapy
25	70	9	II	3.11	3.86	Radical resection
26	58	6	IV	19.11	4.17	Antiandrogen therapy

GS = Gleason Score; SUV = standardized uptake value;

* Based on American Joint Committee on Cancer prostate cancer staging system (2002)
